# Robots as addressable non-persons: an analysis of categorial work at the boundaries of the social world

**DOI:** 10.3389/fsoc.2024.1260823

**Published:** 2024-05-15

**Authors:** Florian Muhle

**Affiliations:** Endowed Chair of Communication Studies with a Focus on Digital Communication, Department of Cultural Studies and Communication Studies, Zeppelin University, Friedrichshafen, Germany

**Keywords:** social theory, membership categorization analysis, non-human agency, communicative AI, humanoid robots

## Abstract

Prompted by the material turn in the social sciences and the development of novel interaction technologies, lively debates in social theory have arisen regarding the agency of non-human entities. While these debates primarily involve exchanging theoretical arguments against the background of different theoretical positions, ethnomethodological membership categorization analysis (MCA) provides an empirical approach to questions of non-human agency. The article discusses the debate on non-human agency, demonstrates how MCA can be used to investigate categorial work at the boundaries of the social, and presents the example of an encounter between two museum visitors and a humanoid robot to show how the robot is categorized in a specific way as an ‘addressable non-person.’ In this way, it becomes clear that social-theoretical debates and empirically oriented MCA can mutually inspire each other and how the ‘basic categorization apparatus’ addresses new alterities.

## 1 Introduction

Ethnomethodology (EM) has always had a complicated relationship with sociological theory. EM’s founders developed it as a strictly empirical approach ‘that rejects top-down theories to understand social structures’ (Nguyen and Nguyen, 2022, p. 261). Ethnomethodologists attempt to gain insights ‘from the data themselves’ (Schegloff and Sacks, 1973, p. 291). They claim to resist so-called ‘academic and theoretical imperialism’ (Schegloff, 1997, p. 165), which does not seriously consider people’s everyday life problems and competencies but rather imposes its own theoretical descriptions, evaluations and categorizations on them. In sociological theory, such a radical empirical approach to social reality was strictly rejected for a long time because it was regarded as ‘aggressively and programmatically devoid of theoretical content of sociological relevance’ (Coser, 1975, p. 696).^1^ Not surprisingly, it has long been difficult for ethnomethodologically oriented researchers to publish their work in sociological journals (Heritage, 2009, p. 300).

Over time, however, EM has gained acceptance in mainstream sociology, establishing itself as a distinct sociological approach that highlights how social order is reproduced as a practical accomplishment within everyday interactions. Simultaneously, EM-oriented researchers have—in a respecified way (Button, 1991)—turned their attention to phenomena that are the focus of classical sociology. EM-oriented research today is not only concerned with ‘ordinary’ everyday life, but also with institutional interaction or the interactional relevance of identity categories such as race, class, and gender (Psathas, 2006). Such categories, however, are not analytic categories from an ethnomethodological point of view but rather ‘categories-in-action’ (Schegloff, 2007b). Interactants use these categories to produce and reproduce social order. Accordingly, social roles in institutional settings and categories of identity are viewed ‘as members’, rather than analysts’, categories’ (Stokoe, 2012, p. 278). In other words, EM-oriented researchers are concerned with tracing the extent to which corresponding categories are made relevant by actors themselves and applied to (re-)produce social order and social differentiations. Membership categorization analysis (MCA) has established a distinct approach for the investigation of corresponding categorial issues (Hester and Eglin, 1997; Schegloff, 2007a; Housley and Fitzgerald, 2009; Stokoe, 2012). This approach was introduced in the early work of Harvey Sacks (1972). However, its role was initially minimal in the EM community, in which conversation analysis (CA) became the most prominent empirical approach (Stokoe, 2012).^2^

In this paper, I resume this line of research to address a topic that has recently gained relevance in contemporary social theoretical discussions, namely non-human agency. In general, I will address the boundaries of the social world and the possibilities and limits of sociality with non-humans, and in particular, I will focus on machines as new alterities. Up to now, existing academic discussions about this topic have primarily been theoretical and often highly normative (Luckmann, 1970; Latour, 2005; Lindemann, 2005, 2021; Knoblauch, 2020). In addition, they had limited connection with existing empirical research. Against this backdrop, I aim to demonstrate that an empirical approach like MCA can promote insight into how the boundaries of the social are drawn in everyday conduct. Hence, MCA can connect existing social-theoretical debates with empirical investigations into the boundaries of the social and thus enrich theoretical discussions. Moreover, the subject area of the hitherto human-centered empirical approach of MCA can be expanded, making it apparent how theoretical considerations and empirical analyses can benefit from each other.

In what follows, I will present a ‘single case analysis’ (Schegloff, 1987) of an encounter between two humans and a robot in a museum. The analysis exemplifies how the boundaries of the social world are (re-)produced *in situ*. Furthermore, it demonstrates how the ‘basic categorization “apparatus”’ (Housley and Fitzgerald, 2015, p. 8) addresses new alterities and how the robot is categorized in a specific way as an ‘addressable non-person’, which appears as a new ‘membership category’ at the boundaries of the social world (section 4). To provide background for the analysis, I will first briefly introduce the social-theoretical discussion regarding the boundaries of the social and its background (section 2). Next, I will present the empirical approach of MCA and its capability for analyzing practices of differentiation between social and non-social entities (section 3). This section will be followed by the single case analysis (section 4) and a short conclusion (section 5).

## 2 Social-theoretical debates about the boundaries of the social world

In modern (Western) societies, ‘common sense permits no doubt that social reality is composed of human affairs’ (Luckmann, 1970, p. 73). That is, modern societies distinguish between a social world of humans and a non-social world, which consists of other entities like machines, animals, and plants. Since sociology is a ‘child’ of modern society, it is not surprising that sociologists normally also ‘equate the social world with the world of living humans’ (Lindemann, 2005, p. 69). Accordingly, they traditionally study only *human* beings who mutually coordinate their actions with those of others, while they are not interested, for example, in the interactions of great apes.^3^ As social theorist Gesa Lindemann puts it,

the field of sociological research is restricted, for example, to the social systems constituted by social actions of living human beings (Parsons), to the symbols developed in human interactions (Mead), or to the actions within human social relationships, which constitute social forms (Weber). (2005, p. 69).

In recent years, however, researchers have questioned this self-limitation of the scope of sociological research. The ‘material turn’ in the social sciences and the emergence of new types of communication technology have challenged the restriction of the realm of the social to humans (Lindemann, 2021, p. 13). In particular, so-called ‘communicative AI’ (Guzman and Lewis, 2020) technologies like ‘social robots’, ‘embodied conversational agents’ (ECAs), and ‘smart speakers’ call the traditional human-centered perspective of sociology into question (Zhao, 2006; Muhle, 2017).^4^ Such technologies are intended to simulate human behavior and may become actors in a way that is traditionally reserved for human beings. Cynthia L. Breazeal, one of the pioneers of social robotics, describes her understanding of social robots as follows:

For me, a sociable robot is able to communicate and interact with us, understand and even relate to us, in a personal way. It should be able to understand us and itself in social terms. We, in turn, should be able to understand it in the same social terms—to be able to relate to it and to empathize with it. […] In short, a sociable robot is socially intelligent in a human-like way, and interacting with it is like interacting with another person. At the pinnacle of achievement, they could befriend us, as we could them. (2002, 1)

Breazeal’s description of social robots emphasizes that the established separation of a social world of humans and a technical world of machines has become blurred with the advent of communicative AI. Consequently, new alterities like humanoid machines have become a relevant subject for sociological inquiry (van Oost and Reed, 2011; Böhle and Pfadenhauer, 2014; Meister, 2014; Mlynář et al., 2018), challenging basic assumptions of sociology. Consequently, new debates have emerged about granting the status of social actors to non-human technical entities, in which traditional ‘humanist’ and new ‘post-humanist’ approaches oppose each other (Muhle, 2018).

In these debates, traditional theoretical approaches still promote a human-centered perspective and deny non-human social agency. Robots and other technical entities are considered tools or ‘objectivations’ of human social activities. In this perspective, represented for example by social constructivists Pfadenhauer and Dukat (2015), communicative AI systems do not engage in social relations with humans. Instead, they remain artifacts that allow for (indirect) relations between humans, namely designers or developers and users (Pfadenhauer and Dukat, 2015). In this line of thinking, machines cannot become social actors and do not have agency. Their activities are pre-programmed and hence were inscribed into the machines by programmers or designers. Even if users grant communicative AI (or other) technologies the status of actors, this status is treated as a ‘projection’, not a social fact (Pfadenhauer, 2015; Knoblauch, 2020, p. 113).

Against this backdrop, post-humanist accounts emphasize the social agency of technical artifacts (and other non-human entities). In this context, actor-network theory (ANT) has become especially influential. ANT explicitly rejects the idea that social relations are restricted to human beings (Latour, 1996; Latour, 2005). On the contrary, proponents of ANT claim that social relations can emerge between all kinds of entities and accordingly ‘extends the word actor—or actant—to non-human, non-individual entities’ (Latour, 1996, p. 369). Following a semiotic definition of ‘actant’, an actor is just ‘something that acts or to which activity is granted by others. It implies no special motivation of human individual actors, nor of humans in general’ (Latour, 1996, p. 373).^5^ Based on this weak actor concept, even a scallop or a door-closer may become an actor in the same way a fisherman or a scientist does (Callon, 1986; Johnson, 1988). Proponents of ANT not only claim that scallops or door-closers *can* become social actors, but that they *should* be treated as social actors in the same way as human beings. According to ANT’s methodological principle of ‘generalized symmetry’ (Callon, 1986), scientific observers *have to* describe the actions of a scallop or door-closer in the same way as the actions of a human being. Callon and Latour express this concept as follows: ‘Whatever term is used for humans, we will use for non-humans as well’ (Callon and Latour, 1992, p. 353).

From an EM-oriented analytical perspective, humanist as well as post-humanist approaches, appear problematic. They both place greater emphasis on the theorist’s analytical perspective than on the differences and similarities between social and non-social entities as they are made relevant by people in everyday social situations. Hence, in terms of Schegloff (1997, p. 167), both humanist and post-humanist social-theoretical approaches reiterate ‘a kind of theoretical imperialism […], a kind of hegemony of the intellectuals, of the literati, the academics, of the critics whose theoretical apparatus gets to stipulate the terms by reference to which the world is to be understood’.^6^ Therefore, from an EM-oriented perspective, another methodical approach is needed—one which allows researchers to investigate the boundaries of the social world and the agency of humans and non-humans as an ongoing interactional accomplishment (Krummheuer, 2015; Pelikan et al., 2022). Such an approach must be sensitive to possible extensions of the realm of the social to non-humans, as well as to the possible differences and asymmetries between entities and their agency that reveal themselves through ongoing conduct (see Suchman, 2007, p. 268–271; Muhle, 2017).

Within the recent social-theoretical debates, Gesa Lindemann advocates for such an approach. Referring to existing anthropological research, she states that the boundaries of the social are (historically) changeable and that ‘in some societies only humans are social actors in their own right, in other societies animals, gods, the deceased, plants, or other things can occupy the status of an actor as well’ (2005, p. 70). The boundaries therefore seem fundamentally contingent on which entities can obtain the status of social actors, an observation that challenges how the boundaries of the social are drawn and how they may change.

Lindemann assumes that whether an entity is considered a social being and, therefore, a potential interaction partner is dependent on a ‘foundational interpretation’ (2005, p. 73). This interpretation is ‘based upon an implicit interpretation that distinguishes those entities whose physical appearance can be seen as an indication of the existence of an entity with which Ego can exist in a [social] relationship’ (Lindemann, 2005, p. 73). In other words, before people enter into a social interaction with another entity, they must recognize whether or not this entity is a social being. Depending on the result of a respective categorization practice, subsequent actions with regard to the other entity will differ. This basic idea is already reflected in Parsons and Shils (1951) concept of double contingency (see also FN 8), which allows for distinguishing

between objects which interact with the interacting subject and those objects which do not. These interacting objects are themselves actors or egos […]. A potential food object […] is not an alter because it does not respond to ego’s expectations and because it has no expectations of ego’s action; another person, a mother or a friend, would be an alter to ego. The treatment of another actor, an alter, as an interacting object has very great consequences for the development and organization of the system of action (pp. 14–15).

Both Lindemann and Parsons and Shils assume that people decide whether they are addressing a social actor based on the physical appearance of their counterpart. However, this method of differentiation of objects can only be implemented if the categorized entities are known and can be directly classified accordingly. Such classification is difficult with new alterities like communicative AI systems, as these are still unfamiliar artifacts for which an appropriate classification is not yet clear. Distinction practices cannot, therefore, simply be based on the physical form, but instead depend on observing the activities and characteristics of the new artifacts. Christian Meyer shares this consideration when he states that people who are confronted with new kinds of alterities apply a basic ethnomethod, which he calls ‘minimal sympathy’ (Meyer, 2016). According to Meyer (2016, p. 93), this ethnomethod implicitly and continuously tests the alterity of the interactional counterpart and uses it to permanently calibrate the ongoing interaction. This can include, for instance, ‘affiliation smiles’ (Martin et al., 2017), tests of language skills and emotions, or tests of the presence of a social ‘face’ (Meyer, 2016).

Without referring to minimal sympathy by that name, some ethnomethodologically oriented studies have already identified corresponding practices in human–machine interaction. Alač (2016), Pelikan et al. (2022), and Rudaz et al. (2023) demonstrate that social robots are sometimes treated as things and sometimes as social actors in human–robot encounters. Similarly, Krummheuer (2008) finds that people often tease their artificial interlocutors in order to explore their communicative capabilities. Therefore, some research in the field of EM already discusses ‘minimal sympathy’ between humans and machines and explores categorization practices in human–machine encounters. However, studies that apply MCA in this research field remain rare,^7^ despite the method’s explicit dedication to the analysis of categorial work in interactions. MCA provides analytical tools for investigating how distinctions ‘between objects which interact with the interacting subject and those objects which do not’ (Parsons and Shils, 1951, p. 14) are drawn *in situ*. In the section that follows, I will further illuminate how MCA can be applied for respective analyses at the boundaries of the social world.

## 3 Membership categorization analysis and the boundaries of the social world

As the term suggests, the main emphasis of MCA involves analyzing the use of membership categorizations during interactions. Such ‘membership categories […] are classifications or social types that may be used to describe persons’ (Hester and Eglin, 1997, p. 3). They are indispensable ‘resources that participants use in interaction with other participants’ (Gafaranga, 2001, p. 1913) in order to develop expectations of their characteristics and predict their next actions, enabling them to mutually coordinate their activities. MCA scholars assume that participants in interactions must categorize their counterparts in order create expectations of their activities, motives, and characteristics. In doing so, people apply and situationally adapt their ‘basic categorization “apparatus”’ (Housley and Fitzgerald, 2015, p. 8), which includes their ‘common-sense knowledge about the world and how social categories are expected or assumed to act in general and in particular situations’ (Housley and Fitzgerald, 2015, p. 8).

This basic apparatus can be understood as a cultural system in operation. It enables people to intuitively categorize their counterparts according to their existing knowledge of the world and treat them accordingly. In particular, this practice facilitates encounters between people who do not know each other and hence require cues to coordinate their actions. In these situations, people start ‘doing everyday sociology’ (Leudar et al., 2004, p. 245) and draw upon their knowledge of the social world. Basically, this means that ‘any person who is a case of a category is seen as a member of a category, and what’s known about that category is known about them, and the fate of each is bound up in the fate of the other’ (Sacks, 1979, p. 13). In this way, people not only show their capability for basic sociological theorizing (Housley and Fitzgerald, 2015, p. 4) but also competently (inter)act and coordinate their behavior in uncertain or rather unfamiliar situations.^8^ Without such a capacity for ‘folk sociology’, people would be unable to engage in interaction and (re)produce society. The task for MCA researchers, then, is to analyze how membership categorization works in practice, in order to build (or rather, reconstruct) the apparatus that makes the observable categorial work possible (Schegloff, 2007a,b, p. 466).

However, it is important to note that categories, which people attribute to each other, are not stable or fixed. Rather, membership categorizations create ‘identities-for-interaction’ (Stokoe, 2012, p. 278). These identities emerge during the course of the interaction and are used *in situ* as resources that allow participants to develop expectations and interpret one another’s actions. Practices of categorization are an essential part of the human ‘interaction engine’ (Levinson, 2020), which organizes interactions in chains and sequences on the basis of expectations (Schegloff, 1968, 2020; Levinson, 2020, p. 45). Therefore, MCA does not treat existing social categories as starting points or explanatory ‘resources’ for social phenomena but as ‘turn generated categories’ (Fitzgerald and Housley, 2002, p. 581). These categories emerge over the course of an interaction, based on the observation and interpretation of observable and interpretable previous actions of the interlocutors. Consequently, membership categories can change during interaction processes. Say, for example, that a person is treated as a ‘punk’ at the beginning of a conversation due to his or her fashion style but then demonstrates knowledge about composers of early 19th-century classical European music. In this case, the same person might be categorized as a ‘Beethoven expert’ in the further course of the interaction. Interaction participants constantly monitor one another’s actions in order to categorize these actions and develop expectations on this basis, which allows for continuing the interaction.

When investigating everyday categorization practices, several analytical concepts are key: (1) membership categories, (2) membership categorization devices (MCDs), (3) category-bound activities, and (4) category-tied predicates. As previously mentioned, membership categories ‘are classifications or social types that may be used to describe persons’ (Hester and Eglin, 1997, p. 3). Membership categories may include ‘sister’, ‘husband’, ‘colleague’, ‘boss’, ‘scientist’, ‘football player’ or ‘musician’. Different kinds of categories trigger particular expectations regarding the properties, typical activities, and expectations of the categorized persons. When talking to a sister, one might expect an informal, warm greeting and her willingness to listen to one’s personal problems. However, at least in Western cultures, one would not expect the same from colleagues or a boss. This example demonstrates how membership categories structure expectations and the course of interactions. Building on the concept of membership categories, the notion of MCDs underscores that certain categories may be linked to form classes or collections. This idea

refers to the fact that, in the locally occasioned settings of their occurrence, some membership categories can be used and heard commonsensically as ‘going together’, whilst others cannot be so used and heard, For example, the collection or MCD ‘family’ may be so heard to include such membership categories as ‘mother’, ‘father’, ‘son’, ‘daughter’, ‘uncle’, ‘aunt’, etc., and to exclude ‘trumpet player’, ‘dog,’ ‘marxist feminist’ and ‘Caucasian’ (Hester and Eglin, 1997, p. 4).

As indicated above, membership categories are inseparable from expectations regarding the particular activities and characteristics of the people who are thought to belong to them. Most people expect a sister to listen to their personal problems, but they do not expect her to apply for the same job as they do. With colleagues, it is probably the other way around. In this context, Harvey Sacks introduced the term ‘category-bound activities’, ‘which are those activities that are expectably and properly done by persons who are the incumbents of particular categories’ (Hester and Eglin, 1997, p. 5). In addition, category-tied predicates consist of ‘attributes, rights, responsibilities, obligations, duties, and knowledges that are viewed as “properly” linked to a category’ (Fox et al., 2023, p. 581). With these four analytical concepts in mind, a researcher’s task is to analyze everyday categorial practices in order to expand knowledge about the underlying categorization apparatus.

Up to now, MCA has been restricted to the analysis of *human* interactions since membership categories are clearly defined as categorical ways of describing or characterizing *persons* (Hester and Eglin, 1997, p. 3; Psathas, 1999, p. 143; Housley and Fitzgerald, 2015, p. 8). Animals, plants, robots, or other kinds of new alterities are not part of this focus. Even though ‘non-personal objects’ (Housley and Fitzgerald, 2002, p. 66) are sometimes considered by MCA scholars, they are only of interest as objects to which humans refer in their interactions. Brooks and Rawls, for instance, write that objects ‘can be oriented toward as stable objects (even though they are constituted), thereby supporting the characterization of object-oriented category membership within human interactional communication’ (Brooks and Rawls, 2012, p. 409). For instance, Housley and Fitzgerald (2002, p. 76) analyze a case in which particular furniture in the context of removal is treated by interactants as belonging to the MCD ‘objects to be moved’.

However, with the advent of communicative AI, the simple ontological distinction between persons and non-personal objects seems to have begun blurring: activities and characteristics that were previously bound to human social actors can potentially also be attributed to machines (see section 2). Hence, it seems sensible to adjust the human-centered perspective of MCA and open its analytical toolbox for open-ended empirical analyses. It has become necessary to consider that machines may be categorized in a similar way as human beings. In order to conduct these analyses, it is essential to recognize that membership categories are not essential properties of the persons being categorized. Hence, membership categories and their connection to particular activities and characteristics are always contingent and undetermined. As Sally and Stephen Hester put it, ‘categories are always “categories in context,” and this means that the task for MCA is to discover how collections, categories and predicates are used on the occasions of their occurrence rather than presuming their stable cultural meanings’ (Hester and Hester, 2012, p. 566). Thus, membership categories are products of everyday interaction processes, not stable properties of humans or other entities.

It therefore follows that categorizations, category-bound activities, and category-tied predicates belonging to particular membership categories or MCDs always underlie transformations that depend on the situated contexts of their use (Hester and Hester, 2012). This observation allows for relaxing the restrictive equation of ‘social actor = human being’ and re-evaluating the distinction between humans and other entities with regard to their belonging to the realm of the social as a (historically instituted) distinction. After all, this distinction may change with the increased occurrence of communicative AI. Seen this way, the established categorical separation of humans and machines in modern (Western) societies can be interpreted as a distinction between two different kinds of MCD, which collect different membership categories along with particular characteristics (predicates) and activities bound to them. In present society, robots, smart speakers, and computers are commonly understood as belonging to the non-social device ‘machines’, a category which is itself part of the collection of ‘non-social entities’. Children, adults, technicians, scientists, and so on belong to the device called ‘humans’, which is used synonymously with the collection called ‘social entities’.

Along with the various MCD affiliations come different expectations regarding the typical activities and attributes of humans, machines, and other entities. For example, if asked, many people would classify humans as living beings who relate to each other, have feelings, and express their emotions. In contrast, they expect machines to function, and assume that machines are not alive and hence do not have feelings that can be hurt or which must be recognized (Edwards, 2018; Fritz, 2018; Guzman, 2020). In line with these ‘ontological’ assumptions about the characteristic predicates and activities of humans and machines, people interpret humans as belonging to the MCD of ‘social entities’, while this is not the case for machines.

However, there is no reason this categorization should not change if machines attain the capability to ‘interact meaningfully with humans’ (Morie et al. 2012, p. 100), as their developers intend (see section 2). Typical activities that are currently limited to human beings may then be performed by robots and other communicative AI technologies. There is already evidence that the fundamentally different categorization of humans and machines is beginning to falter. For example, people are beginning to ascribe emotions to communicative AI systems (Pelikan et al., 2020). As communication scholar Andrea L. Guzman puts it,

many of the ontological boundaries […] remain lines of delineation between people and computers from the perspective of the public. Some of these divides, however, are no longer as clear as they once were or are becoming even more complex. Most people consider emotion to be a key boundary, but some people’s interactions with communicative technologies designed to emulate human emotions, such as Apple’s Siri, have caused them to reassess the degree to which emotion remains a human trait (Guzman, 2020, p. 50).

It is precisely this situation, in which the boundaries between man and machine have become blurred, that renders the ethnomethod called ‘minimal sympathy’ (see section 2) significant. ‘Minimal sympathy’ enables people to make sense of an artificial counterpart’s actions and develop expectations that help to structure unfamiliar encounters with new alterities. Accordingly, ‘minimal sympathy’ functions as a basic method of the categorization apparatus that aims to categorize new alterities based on tests of their predicates and activities. Against this backdrop, it appears plausible to open the analytic toolbox of MCA for analyses that explore the everyday (re)production of the boundaries of the social world, in order to expand knowledge about the categorization apparatus and members’ methods for addressing new alterities like communicative AI systems.

For this analysis, it is sufficient to follow the sequential course of boundary situations and explore whether (and if so, how) participants determine different kinds of categorizations step-by-step as ‘turn-generated categories’. In this way, it is possible to ‘reconstruct the ways in which a social position is introduced, maintained, and eventually suspended turn-by-turn’ (Hausendorf and Bora, 2006, p. 88) and, hence, to study the working of the categorization apparatus in encounters with new alterities. The leading empirical question is, therefore, how participants in concrete encounters with new alterities categorize each other and whether the alterities are granted predicates that, up to now, have been reserved for humans. If machines (or other non-humans) are attributed behaviors and characteristics typically associated with social beings, significant transformations regarding the realm of the social are potentially occurring; in this case, the ontological human/machine difference as one of the demarcation lines between social and non-social actors (see sections 1 and 2) becomes fragile.

The next section addresses how analyses of categorial work at the boundaries of the social world can be conducted and what insights they might reveal. It presents a single case analysis of the beginning of an encounter between two humans and a humanoid robot in a computer museum. The analysis provides detailed insights into the sequential unfolding of members’ ‘categorical ordering work’ (Hester and Eglin, 1997, p. 3) at the boundaries of the social world.^9^

## 4 Single case study: the sequential unfolding of categorizations in an encounter with the humanoid robot ‘Nadine’

**Figure 1:**
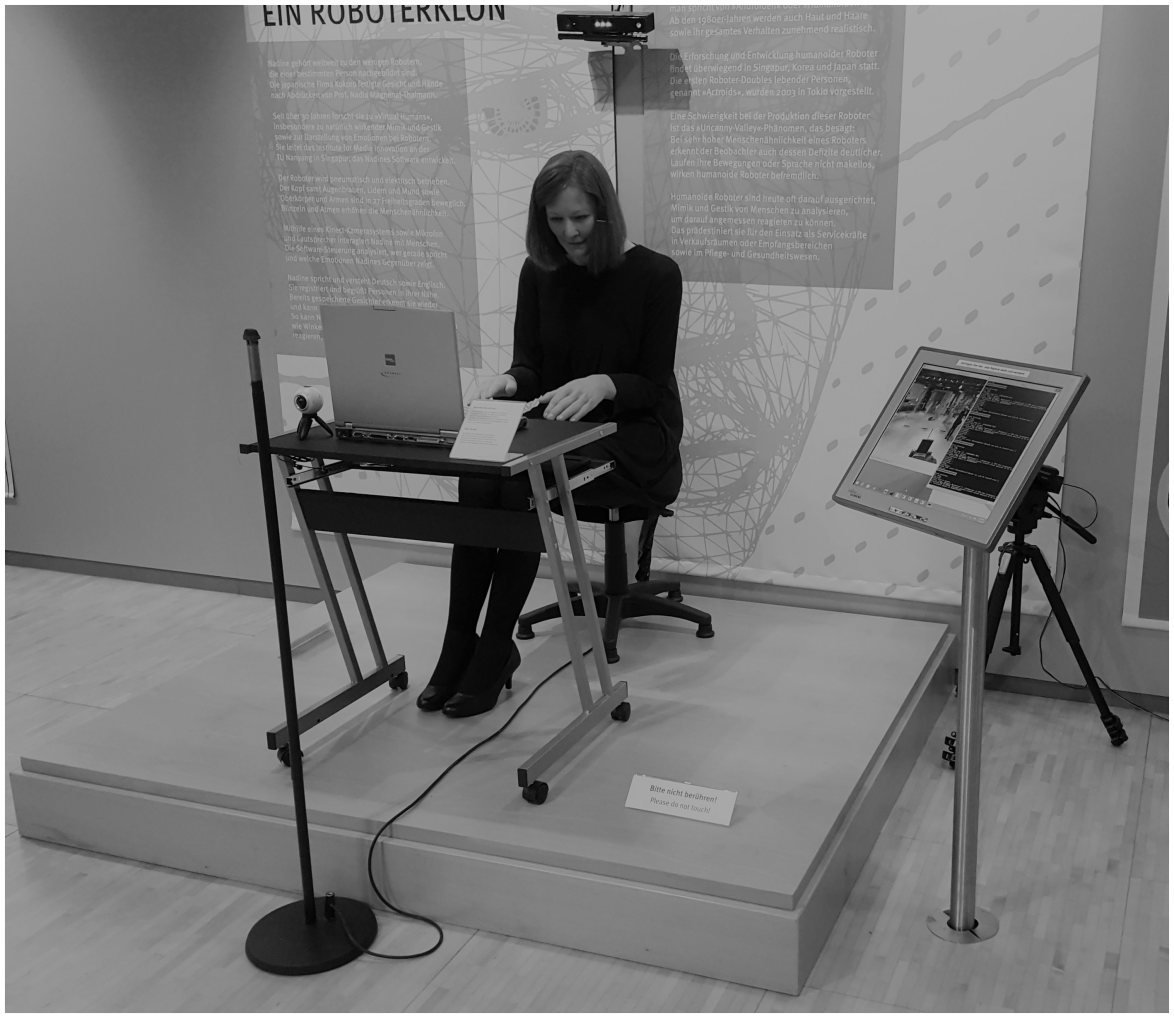
The robot in its/her basic posture.

**Figure 2:**
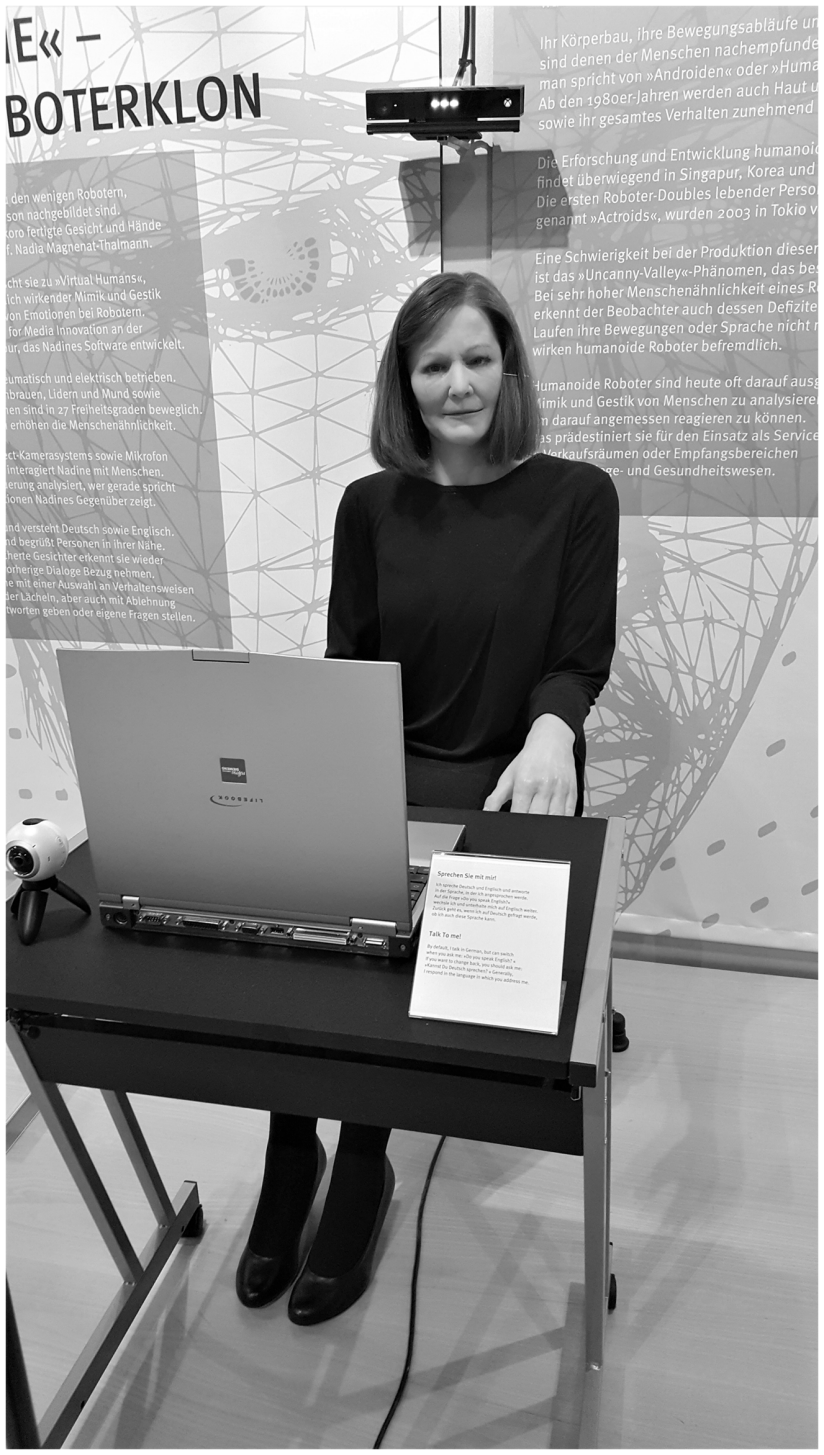
The robot in its/her interaction posture.

The data that serve as a basis for the following analysis stem from the research project ‘Communication at the Boundaries of the Social World’, which explored the possibilities and limits of the social agency of new interaction technologies.^10^ In the context of the project, several encounters between museum visitors and different communicative AI systems were videotaped, transcribed, and analyzed. One of the technical systems under investigation, which is also the subject of the following single case analysis, is the humanoid robot ‘Nadine’. The robot looks like its/her^11^ developer, Nadia Magnenat Thalmann, and has a remarkable human resemblance. Nadine is a ‘sitting pose robot’ (Magnenat-Thalmann and Zhang, 2014, p. 4) that can move its upper body, arms, and head (see Figures 1, 2). Additionally, the robot performs rudimentary facial expressions and gestures. It/she is able to recognize people (faces as well as some gestures) and provides a voice user interface.

According to its developers, Nadine, like other social robots, is meant to serve as a companion with whom one can communicate in an intuitive and natural manner (Magnenat-Thalmann and Zhang, 2014). In the museum, the robot is placed on a small pedestal, in a sitting position, behind a desk with a laptop on it. On the wall behind Nadine are two information boards. On one board, Nadine is introduced as a ‘robot clone’, and its/her mode of operation is explained. Among other things, its/her ability to move and its/her modes of propulsion (electric and pneumatic) are described, as well as its/her ‘perception apparatus’, which consists of a Kinect camera system, a microphone, and a loudspeakers and enables her to talk with people in a technically mediated way. On the other information board, museum visitors find general information about the history and subject of research on humanoid robots. In addition, on the desk in front of Nadine, a small sign states, ‘Talk to me!’ and informs the reader that Nadine understands and can speak both German and English. On the floor of the pedestal, there is another sign with the request ‘Please do not touch’. Nadine is thus presented on the one hand as a human-like robot that can engage in conversations with visitors via a technical interface, but on the other hand, as an exhibit that is the object of reception that—unlike typical interactive exhibition objects (Heath and vom Lehn, 2008)—may not be touched.

In its/her ‘basic posture’, the robot sits slightly slumped in the chair with its/her gaze directed at the computer. The arms are bent in front of the body as if the robot were typing (see Figure 1). The camera system that serves as ‘eyes’ is mounted in the background above Nadine’s head, and the microphone that allows museum visitors to make auditory contact is located in front of the desk. As soon as the camera system registers movement in Nadine’s environment, the robot raises its/her upper body and head and directs its/her gaze in the direction of identified possible interaction partners. After successful identification, Nadine usually greets these partners in a nonverbal way, by waving, and Nadine assumes its/her ‘interaction posture’ (see Figure 2).

The museum visitors almost inevitably encounter Nadine when they enter the exhibition space. Thus, they are normally registered by Nadine’s sensors and subsequently confronted with the robot’s attempt to make contact. Occasionally, the visitors ignore the attempt and continue to the next exhibit. Most visitors, however, are at least interested enough in Nadine’s movements to approach the robot, stop in front of the desk, and engage in interaction with it/her. Only 19 out of 203 recorded encounters did not result in a verbal exchange. This means that in more than 90% of cases, users made an attempt to engage in interaction.^12^ This was the case in the situation that will be analyzed in this paper. The following analysis demonstrates the peculiarities of categorial work in encounters with the humanoid robot.

**Figure:**
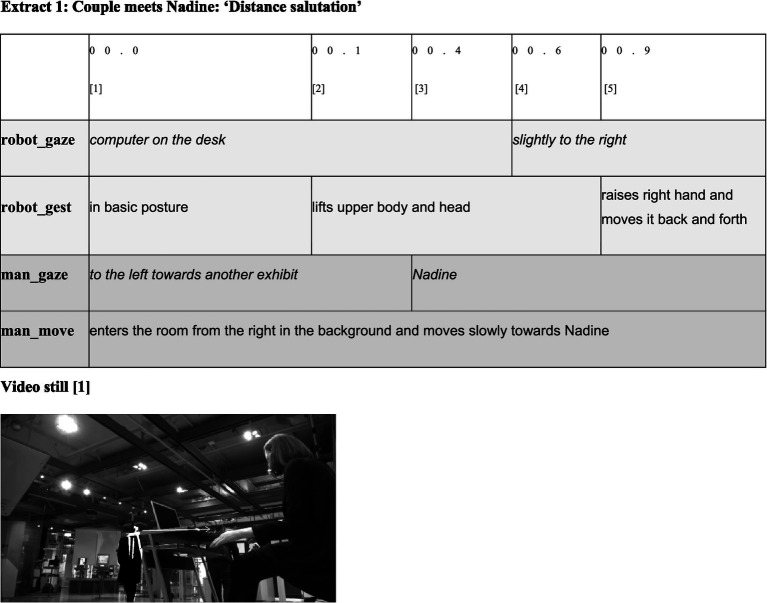


The encounter was recorded with two video cameras and transcribed multimodally using the computer program ELAN, which was developed by the Max Planck Institute for Psycholinguistics in Nijmegen. Since ‘actions [and therefore also categorization practices; F.M.] constituting sequences are not exclusively performed through practices of speaking, but can also be accomplished via embodied practices’ (Evans and Fitzgerald, 2017, p. 82), in addition to the spoken word, gaze direction, facial expressions, gestures, and movements are transcribed as well (see also Mondada, 2007, 2014; Goodwin, 2010, 2018). In order to consider these modalities in both their simultaneous and sequential production, the transcript follows a partiture representation in the form of a table. The simultaneously produced activities of the respective participants are noted on separate lines. For enhanced clarity, the lines assigned to the participants are highlighted in the same shade of gray. Gaze directions appear in italics, while gestures and facial expressions are noted in regular text. The verbal exchange is transcribed according to the conventions of the GAT2 transcription system (Selting et al., 2009).

The sequential nature of the multimodal events becomes clear when the tables are read from left to right. The first line shows the time stamps at which the various activities begin. In addition, the columns in the first line are numbered so it is possible to refer by number to the activity beginning at the specific point in time. The duration of activities can be recognized by the length of the cells in which they are noted. Every table is supplemented by a still from the video recording so it is possible for the reader to visualize the situation.

The encounter under investigation lasts just under 2.5 min. The analysis, however, covers only the first 33 s. Nevertheless, this time frame is sufficient for gaining detailed insights into the ongoing categorization processes, which is unsurprising since the (re)constitution of the relationship between the participants is a key task in conversation openings (Schegloff, 1986).^13^ That is, members start to categorize each other immediately, in terms of being able to relate to each other and developing expectations. I will analyze the sequential unfolding of the situation in nine steps.

As extract 1 reveals, the situation begins in a similar manner to typical human encounters in public (Kendon, 1990). Immediately after a man enters the exhibit room [1], Nadine raises its/her head and upper body [2] and starts to wave its/her hand [5]. That is, the robot leaves its/her basic posture and moves into the interaction posture. This activity indicates that Nadine has recognized movements in its/her environment and thus shows characteristics (category-bound predicates) of a living being that can recognize its environment and react accordingly. In addition, the hand movement can be interpreted as a ‘distance salutation’ (Kendon, 1990, p. 159) in the context of the ‘pre-beginning’ (Schegloff, 1979) of an interaction. In this sense, in terms of MCA, the robot’s embodied activities can be interpreted as a social action (i.e., an activity bound to the MCD ‘social entities’). Simultaneously, the robot’s gesture indicates that the man is treated as belonging to the MCD ‘social entity’ as well. Otherwise, it would not make sense to provide a distance salutation. Accordingly, Nadine’s hand movement can be understood as an offer to enter a social interaction between two entities that belong to the MCD ‘social entities’, which can engage in social interaction.

**Figure:**
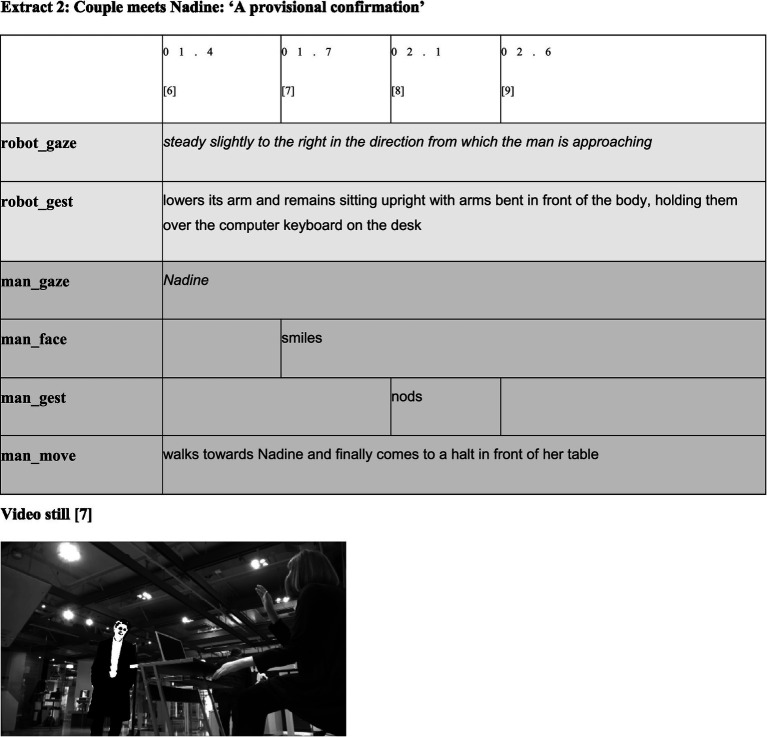


However, membership categorization is a sequential process in which membership categories are not merely introduced. They must be maintained and can also be rejected and transformed; this categorization requires confirmation in the next step. As extract 2 demonstrates, this confirmation seems to occur.

As the man’s behavior in extract 2 shows, the robot’s categorial offer seems to be accepted. After having recognized Nadine [3, extract 1] and established mutual recognition, the man walks toward the robot [6], begins to smile [7], and nods briefly [8]. In performing respective activities, the man, like the robot, not only shows typical predicates of a living being (being able to recognize other entities) but also performs reciprocal actions typical for the beginning of interactions (Schegloff, 1968; Kendon, 1990). In doing so, he indicates both his capability and willingness to engage in interaction and confirms the robot’s offer to treat both as belonging to the MCD ‘social entities’. Simultaneously, however, the man’s smile can, according to the considerations on the ethnomethod of ‘minimal sympathy’ above (sections 2 and 3), also be interpreted as a test of the social capabilities of the robot to find out how to adequately categorize it. That is, the smile at the given sequential position potentially only indicates a provisional confirmation of the robot’s categorization as a social entity, which could be challenged and possibly changed in the following turns.

If the participants subsequently act in accordance with expectable activities of social entities in the context of an interaction opening, they may next exchange ‘close salutations’ and then enter a focused interaction (*cf.* Kendon, 1990: 191ff). If they do so, they confirm their attributed categorial status. If they act differently, however, the categorization apparatus must adapt to this situation and apply categorizations that might fit better to make sense of what happens next.

**Figure:**
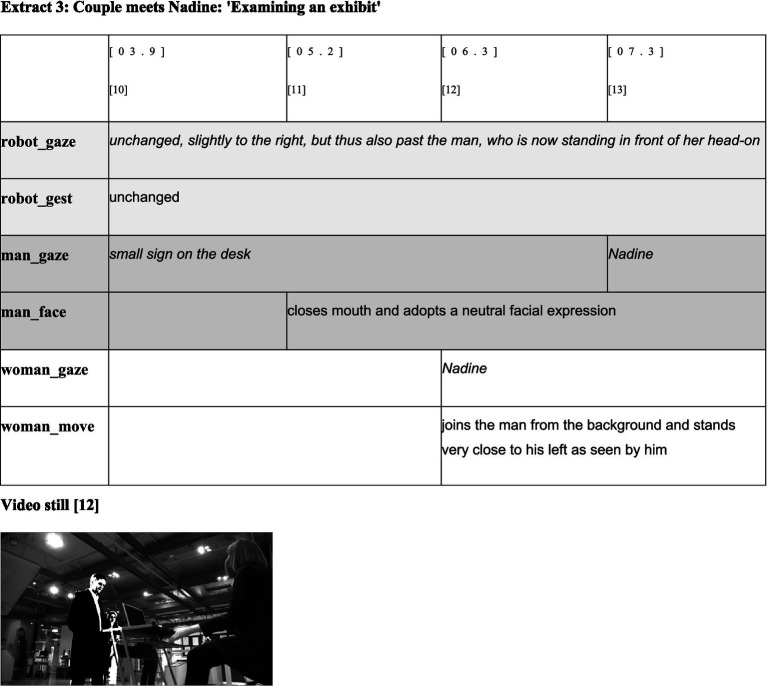


As extract 3 reveals, neither a close salutation nor any kind of verbal exchange takes place. That is, the situation does not proceed in accordance with common expectations in the context of a ‘normal’ interaction opening (Schegloff, 1968; Kendon, 1990). Regarding the robot’s activities, it is striking that both gaze and gesture remain unchanged [10]. The robot’s movements freeze, so to speak. As a consequence, Nadine no longer looks at the man but past him, as he has moved to the desk in the meantime rather than remain where he was initially perceived. This means that the robot’s perception stops, and its/her activities no longer appear to be coordinated with those of the man. The ‘freezing’ of the activities also means that the impression of liveliness becomes lost. Instead, the robot assumes the attributes of an inanimate object, jeopardizing the previously offered predicate of a living being capable of perception.

In light of the conspicuous ‘absent activities’ (Stokoe, 2012, p. 281) on the part of the machine, it is not surprising that the man’s activities, for their part, are no longer interpretable within the context of an interaction opening. Simultaneously with the freezing of the robot’s activities, the man redirects his gaze and looks at the small sign on the desk instead of at the robot. Shortly afterwards, he also stops smiling and closes his mouth [11] before directing his gaze back at the robot—but now with a neutral facial expression [13]. In performing embodied activities, the man—in contrast to the robot—still acts as a ‘social entity’. However, he no longer acts as a potential ‘interaction partner’ of the robot, but rather as an incumbent of the membership category ‘museum visitor’ who engages in activities typical for the examination of exhibits (Heath and vom Lehn, 2004; vom Lehn, 2006). His activities indicate that he is now attempting to make sense of the exhibit by using additional ‘semiotic resources’ (Goodwin, 2000), such as by reading the information signs associated with the object and looking closer at the robot, which/who is now observably viewed as a ‘watchable’ museum object.

In the meantime, a woman enters the scene, looking at the robot as well [12]. By positioning herself very close to the man and thus entering his ‘personal space,’ she indicates that they belong together and are visiting the museum together.^14^ This action changes the situation, as the arrival of the woman now creates space for interaction between the two humans, and the man is no longer alone with the robot.

**Figure:**
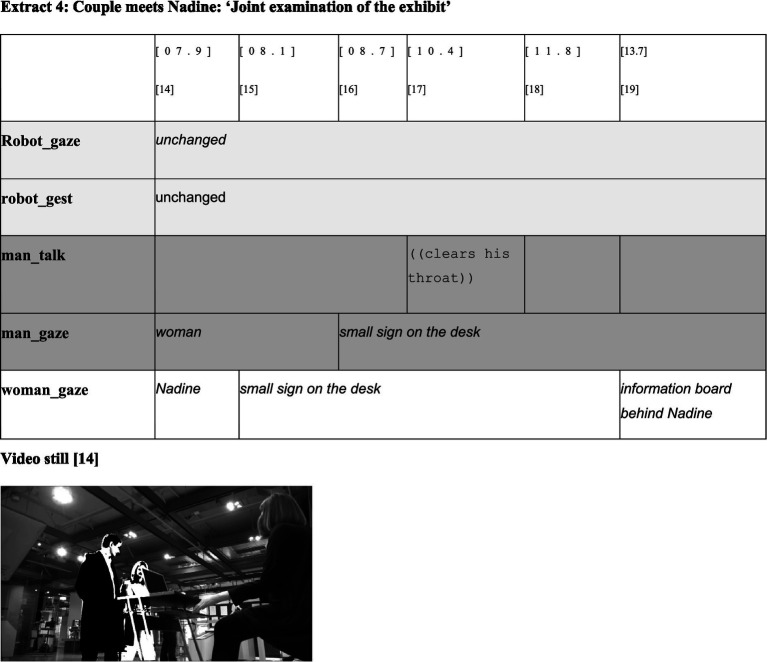


The man’s gaze in the woman’s direction indicates that he recognizes her [14]. By not stepping aside, he confirms that they belong together; she is not invading his personal space but is allowed to enter it. Like the man did before, the woman directs her gaze to the sign on the desk [15] and then to the information board behind Nadine [19]. As the man’s gaze follows the woman’s gaze to the sign on the desk [16], some kind of synchronization of their activities becomes visible. This behavior confirms that they act together in a typical manner as joint museum visitors, engaging with a watchable museum object and information that is provided about the exhibit (Heath and vom Lehn, 2004; vom Lehn, 2006). Accordingly, in this sequential position, Nadine is no longer categorized as a potential interaction partner but as a watchable museum object. Since the robot itself/herself remains passive and thus displays predicates of an inanimate object [14], this categorization is confirmed in this moment of the encounter.

While examining the exhibit, the man clears his throat [17]. According to Schegloff (1996), this kind of activity can be characterized as a typical ‘pre-beginning element’, which could project ‘the beginning of a (next) TCU or a turn’ (Schegloff, 1996, p. 92) but is not yet a proper beginning^15^. This sound might indicate that he starts to provide a turn, which will be directed toward the woman as part of their common museum visit (Heath and vom Lehn, 2004, pp. 49ff). Presumably, he will thematize the robot as a museum exhibit. As the next extract shows, this expectation was partially fulfilled.

First, the woman and the man proceed to visually examine the robot and its/her environment [20–22] and, thus, they continue activities bound to the membership category ‘museum visitor’. Then, about 4.5 s after clearing his throat, the man starts a turn, which is directed to the woman. He says quietly to her that he does not know what to ask [23]. In doing so, he talks *about the robot* while also indicating that he is willing to talk *with the robot* but has no clue for how to start. This means that the man is facing a fundamental problem in terms of conversation opening, since ‘if there is to be a conversation it must be about something’ (Schegloff, 1968, p. 1092). Not knowing what to ask means that he finds himself in a ‘completely indeterminate situation’ (Vanderstraeten, 2002, p. 84), and, hence, in a ‘pure’ double contingency (Luhmann, 1995, 103ff) that makes it impossible for him to address a question toward Nadine and enter a focused interaction with the robot.

**Figure:**
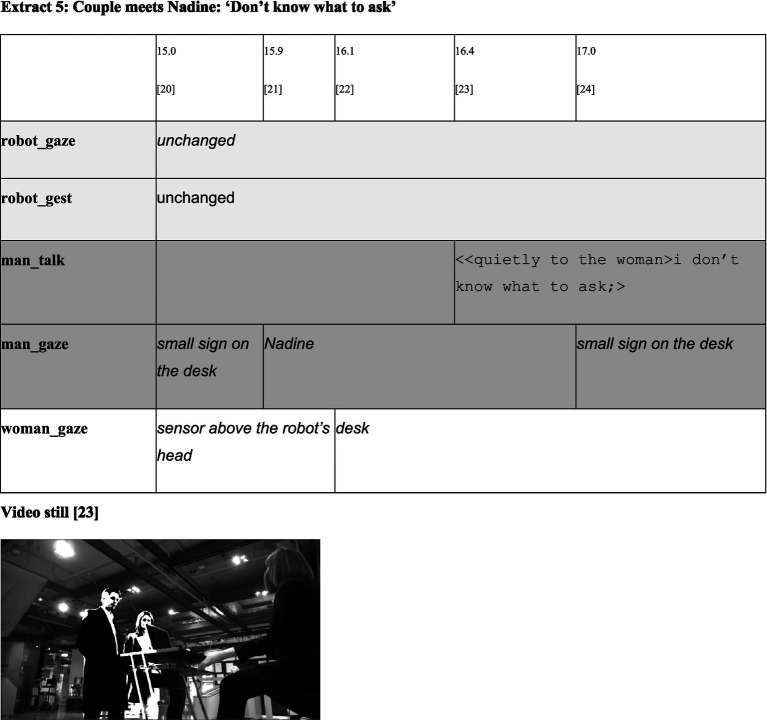


By disclosing this challenge to his companion, the man observably proceeds to act as an incumbent of the membership category ‘museum visitor’, who now talks with his companion about the examined exhibit and thereby engages in shared object reception. The content of his utterance, however, suggests that simultaneously, he remains oriented to the possibility of engaging in interaction with the robot but is not able to solve the problem of how to begin this interaction. In this way, the sequence, in which he (together with the woman) examines the exhibit and searches for additional ‘semiotic resources’ in its surroundings, can be regarded as an insertion that interrupts the opening of a focused interaction with the robot. He seeks clues to a possible topic of conversation between two entities who are ‘maximal strangers’ (Anton and Schetsche, 2023, p. 12) to each other. Meeting such a maximal stranger means that the man is—at this moment—unable to categorize his counterpart more precisely and develop assumptions about the typical characteristics, activities, or attributes of Nadine that could help him to find a possible common topic. Regarding predicates that he attributes to Nadine, this means that, on the one hand, he assumes the robot, in principle, is capable of understanding and answering questions and hence is a potential interaction partner. On the other hand, however, this potential interaction partner seems so strange that it appears too difficult at this moment to experiment with any kind of question to determine what can happen next.

In the second after the man’s utterance, both humans continue to visually examine the robot and its/her environment. Immediately after discovering the microphone in front of the desk [27], the woman steps closer to it and leans over it [28]. In doing so, she indicates that she is now willing to talk to the robot, while the man follows her movements and looks at the microphone after the woman leans over it [29]. She takes over the role of participant in the human–robot encounter and visibly prepares a first verbal utterance, which will be directed toward the robot. In performing these actions, she confirms the categorization of the robot as a possible interaction partner and marks the beginning of the attempt to enter into a focused interaction. This supposition is supported by the fact that the woman also directs her gaze toward Nadine and thus addresses it/her visually [30]. In response, the robot begins moving again for the first time after a period of inactivity. It/she is observed returning the woman’s gaze, which once again creates the impression of mutual perception [31]. Consequently, the requirements for beginning a focused interaction appear fulfilled.

**Figure:**
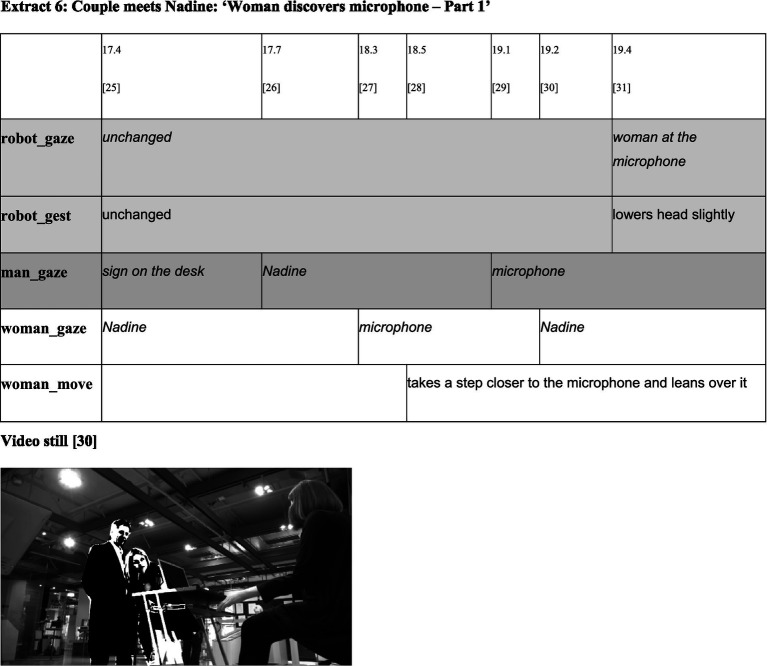


The woman assumes the task at which the man previously failed. She asks the robot a question [32]. Then, the woman returns to her previous upright position [35] while smiling at the robot and thus signaling it/her to take the next turn [36]. The woman’s initiative is affirmed by the man, who signals his approval with a smile [33].

By asking a question, the woman explicitly confirms the assumption that the ability to interact and respond is a predicate of the robot. However, compared to typical beginnings in ordinary human interactions, her utterance is striking in two respects. First, it is noticeable that the woman refrains from expressing a close salutation, which would be expected as part of the pre-beginning of typical social encounters (Kendon, 1990, 191ff)^16^. Second, the choice of topic is unusual. Under normal circumstances, beginning an interaction with a question about age risks violating the ‘face’ (Goffman, 1967) of the counterpart and thus would be experienced as a ‘face-threatening act’ (Brown and Levinson, 1987, 65ff). Even in a conversation with a child, to whom questions about his or her age are in principle legitimate and quite common, such a question would hardly be expected without a preceding salutation and establishment of some kind of mutual relationship. The chosen beginning is thus atypical and violates basic norms of politeness that apply in ordinary conversations. It conveys ‘that the speaker does not care about the addressee’s feelings, wants, etc.’ (Brown and Levinson, 1987, p. 66).

In line with Krummheuer’s (2008) finding that in human–machine encounters, people often tease their artificial interlocutors in order to explore their communicative capabilities, the question can be understood as an application of the ‘minimal sympathy’ ethnomethod, through which predicates of the robot are being tested^17^. Failing to treat Nadine as an Alter Ego with feelings that can be hurt, is, in this sense, a test of whether the robot accepts this treatment; thus, the woman’s utterance confirms the absence of ‘face’. At the same time, however, the woman treats the robot as an entity that can participate in interaction and understand its normative implications. Asking a question makes an answer conditionally relevant (Schegloff, 1968, 1083ff), implicitly expressing the assumption that the addressee is familiar with normative rules of everyday interaction and is capable and willing to act according to these rules.

**Figure:**
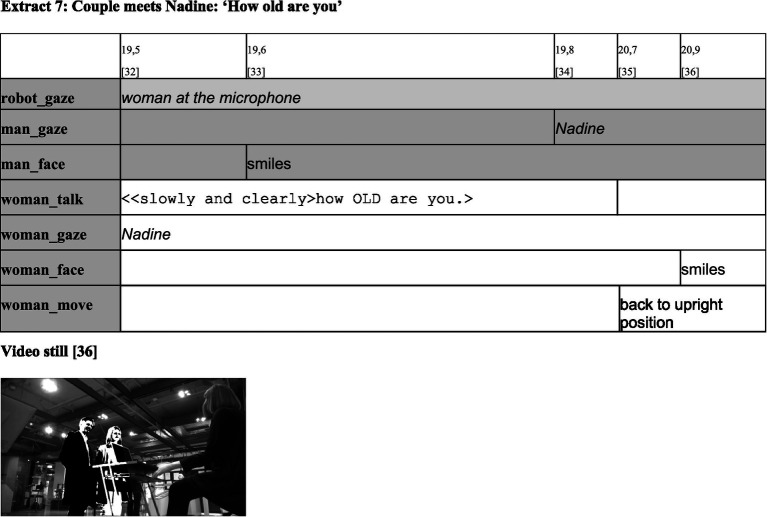


**Figure:**
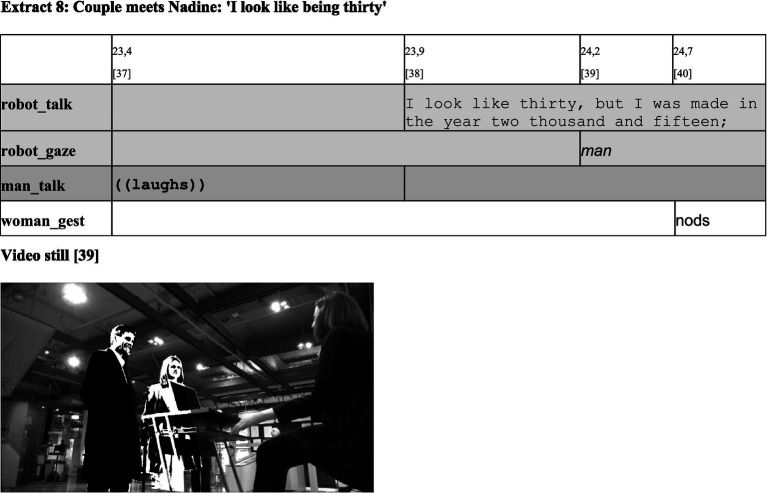


**Figure:**
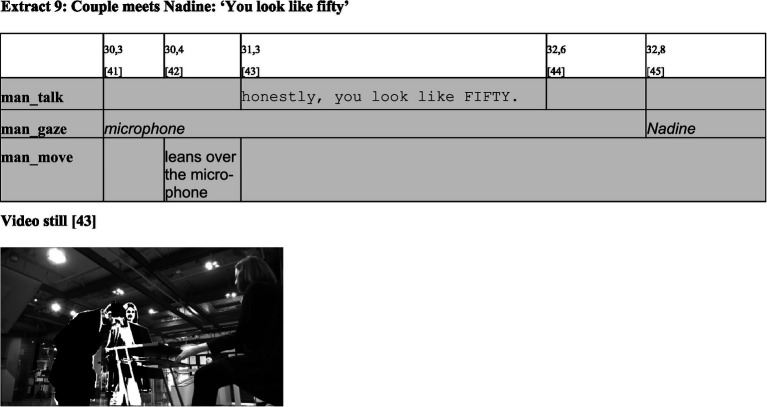


In a typical interaction between incumbents of the MCD ‘social entities’, one would expect Nadine to defend its/her moral status and in doing so, to threaten the others’ faces (Brown and Levinson, 1987). If she did so, she would resist the membership categorization as an addressable entity without feelings and face, instead insisting on an identity as a social entity with respective predicates. As the next extract shows, this is not the case.

First, the man affirms the proposed categorization of the robot. He is not ashamed or indignant in his reaction but instead laughs after the woman has asked her question [37]. This behavior indicates his agreement with treating the robot as an addressable non-person. Then, Nadine responds without expressing offense [38]. Instead, the response turns out to be humorous, since the robot states that it/she appears to be age 30, although it/she is modeled after computer scientist Nadia Magnenat Thalmann, who was older than 60 at the time of Nadine’s development. At the same time, Nadine does not hide its/her robot status, since it/she reveals that it/she was manufactured in 2015. The robot thus presents itself/herself as a technical being that is able to participate in communication and can even show a human—namely humorous—side. Nevertheless, Nadine does not claim to be categorized as a person with feelings—as anticipated by the woman’s question. By answering this prompt, the robot reacts in accordance with the normative expectations of everyday conversations, which make an answer to the preceding question conditionally relevant. However, the content of the question is not regarded as a face-threatening act, which means that Nadine is not insisting on having face but rather accepting the way the woman treats it/her. In this way, the robot confirms its/her categorization. This gives the encounter a strong asymmetry. It prompts a categorization of the participants according to which only the woman possesses the category-tied predicate ‘face’, which needs to be respected. Meanwhile, the robot is lacking face and hence is categorized as a particular kind of ‘non-person’ that is physically present but lacking personhood (Goffman, 2022 [1953], to indicate that the book originally was written in 1953 p. 84). Therefore, no expectations or demands on the side of the robot must be taken into account.

Nadine’s response is acknowledged by the woman, who nods, while Nadine is finishing its/her utterance [40]. Consequently, two membership categories have been established, which can serve as a basis for the subsequent interaction between social persons on the one hand and a robot that can communicate without being a person on the other hand.

In what follows, the man builds upon this established structure and takes the floor to produce his first utterance, which is directed to the robot. With his response—‘Honestly, you look like fifty’ [43]—he comments on Nadine’s preceding answer in a way that—like the woman’s earlier question—would be considered a face-threatening act in typical conversations between incumbents of the category ‘person’. In giving this response, he confirms the categorization of the robot as an *addressable non-person.* Hence, he contributes to the reproduction of the established membership categories and their predicates.

My analysis ends here, as no significant new insights regarding the categorization of the participants emerge in the further course of the encounter. Humans and robots exchange a few more questions (e.g., about their names, their birthplace, and their favorite films), in the course of which the museum visitors make some remarks that would be considered condescending toward incumbents of the category ‘person’. These remarks, however, are again not problematized by the robot, and finally the two visitors leave the scene without saying goodbye.

## 5 Conclusion

In this single case analysis, it is evident that during the encounter, the robot Nadine is assigned a specific membership category, which I have termed ‘addressable non-person’ in reference to Goffman (2022[1953])’. This new membership category at the boundaries of the social world, however, must be distinguished from the non-persons Goffman described. He assigned foreign travelers, who did not understand the language of the locals and could therefore be treated as absent despite their physical presence, to this category. However, the robot’s situation is different. The humanoid robot Nadine, as a new kind of artifact, understands the language of the ‘natives’ but is not a human being. As an artificial interaction partner, the robot is ascribed the ability to participate in interaction in a basic and sometimes even creative—namely humorous—way. A typical activity in this context involves answering questions from human conversation partners. It is expected that the robot can understand these questions and answer them appropriately, and it/she does so. Accordingly, the robot is expected to act according to basic norms of interpersonal interaction. Conversely, this response is not obligatory for humans in relation to the robot. They do not have to consider possible emotions or ‘face’ on the part of the robot. Such qualities are therefore not attributed as predicates of the membership category ‘addressable non-person’. That is, Nadine obviously does not possess the same rights—and thus, the category-tied predicates—that are usually accorded to human beings who are treated as persons.

The empirical analysis demonstrates that the advent of new alterities leads to new forms of categorizations that are not absorbed into established subject/object distinctions. In the case studied, the humanoid robot Nadine is categorized neither as a person nor as a stable and passive object in a classical sense. Instead, the robot is assigned characteristics that stem from both worlds. By taking into account theoretical debates on non-human agency (see section 2), it was possible to explore this categorization and demonstrate how the empirical view can contribute to questioning established dichotomies. While partly irreconcilable basic positions and assumptions clash in the theoretical discourse, technology users must address the question of how to interact with non-humans when they encounter social robots and other forms of communicative AI in practice. As the analysis has shown, their ‘folk sociology’ leads to more diverse results than the specialist sociological debate. The human participants in the encounter do not attempt to fundamentally clarify the robot’s identity once and for all. Instead, in each ensuing moment, they have the opportunity to reconsider and reproduce the situation in its constitutive details, and thus to refine the robot’s categorization. It is therefore appropriate for sociological theorists to orient their theoretical apparatus, to a greater degree, toward the basic categorization apparatus of the social practice and sharpen it accordingly.

MCA can serve as a productive tool in this endeavor, as it aims to systematically reconstruct this apparatus with precision. In the empirical analysis (section 4), the viability of the ‘minimal sympathy’ (section 2) ethnomethod became evident as a means of testing characteristics of new alterities and categorizing them adequately. Further research must investigate and incorporate this method further and render it visible as an elementary component of the basic categorization apparatus in all its manifestations. Undoubtedly, this method’s relevance will increase as new alterities emerge.

## Data availability statement

The original contributions presented in the study are included in the article/supplementary material, further inquiries can be directed to the corresponding author.

## Author contributions

FM: Writing – review & editing, Writing – original draft.

## Footnotes

^1^Conversely, EM was not trying to criticize sociology. Instead, most EM-oriented scholars were indifferent toward sociology’s concerns and interests (Garfinkel and Sacks, 1970).

^2^In addition to CA and MCA, there are also other empirical lines of research within EM, such as workplace studies or praxeological studies.

^3^For an exception, however (see Servais, 2013).

^4^In addition, ethnological and anthropological work shows that not all societies have historically limited the realm of the social to human entities (Luckmann, 1970; Lindemann, 2021).

^5^Similarly, Karen Barad, a proponent of feminist new materialism writes that ‘in traditional·humanist accounts, intelligibility requires an intellective agent (that to which something is intelligible), and intellection is framed as a specifically human capacity. But in my agential realist account, intelligibility is an ontological performance of the world in its ongoing articulation. It is not a human-dependent characteristic but a feature of the world in its differential becoming’ (Barad, 2007, p. 149).

^6^Ironically, the proponents of ANT emphasize that it is important ‘to follow the actors themselves’ (Latour, 2005, p. 12) and their interpretations of the world, similar to the EM approach. However, the principle of generalized symmetry systematically obstructs this attempt.

^7^Exceptions are Krummheuer (2016) and Muhle (2017).

^8^From a social theoretical perspective, the use of membership categories can be interpreted as a members’ method for confronting the fundamental social problem of double contingency (Parsons and Shils, 1951; Luhmann, 1995) Double contingency means that in social interactions, both interlocutors experience that the behavior of the alter ego is unpredictable and capable of variation. Each is a ‘black box’ for each other.

^9^The analysis will show, if and in which sense not only the humans but also the robot can also be understood as ‘member’. This would be the case, if the robot is attributed behaviors and characteristics typically associated with social beings (see above), and hence it/she would be treated as a social entity that is able to participate in social interaction.

^10^The project was funded by the German Research Foundation (DFG) and led by the author from 2016 to 2021.

^11^Since the categorial status of the robot is ambiguous, I refer in my analysis to the robot as it/she. I owe this idea to a reviewer of the article, whom I would like to thank.

^12^What happens next varies. In some cases, the visitors do not interact with Nadine but simply look at her as they look at other objects in the exhibition. Nadine is thus not categorized as a person, and the robot remains socially excluded as a non-personal object, which may become topic of conversations between visitors. In many cases, however, attempts to reciprocate the contact begin. As a rule, however, this proceeds differently than in comparable interpersonal interactions.

^13^Without referring to membership categorization practices, Pitsch et al. (2009) argue that in human–robot encounters, the opening phase is key for establishing and maintaining user engagement.

^14^Otherwise, she would have kept some distance.

^15^TCU is an abbreviation for ‘turn construction unit’.

^16^In our data collection, visitors refrained from a salutation in about 40% of the cases. This indicates that greeting a robot is regarded as possible but not obligatory.

^17^In our data collection, however, this occurred in less than 20% of the cases. In these cases, Nadine was often asked about her age. Additionally, she was sometimes insulted or asked for a date.
